# Evaluation of balance functions using temporo-spatial gait analysis parameters in patients with brain lesions

**DOI:** 10.1038/s41598-021-82358-2

**Published:** 2021-02-02

**Authors:** Byung Joo Lee, Na-Young Joo, Sung Hyun Kim, Chung Reen Kim, Dongseok Yang, Donghwi Park

**Affiliations:** 1 Department of Rehabilitation Medicine, Daegu Fatima Hospital, Daegu, Republic of Korea; 2 Department of Physical Medicine and Rehabilitation, Ulsan University Hospital, University of Ulsan College of Medicine, 877 Bangeojin sunhwando-ro, Dong-gu, Ulsan, 44033 Republic of Korea

**Keywords:** Biophysics, Neurology

## Abstract

This study aimed to compare gait analysis and balance function measurements, such as the Berg balance scale (BBS) score to seek specific measurements that can represent the balance functions of patients with brain lesions. Additionally, we also compared other different gait function scale scores with gait analysis measurements. This study included 77 patients with brain lesions admitted to our institution between January 2017 and August 2020. Their gait analysis parameters and clinical data, including personal data; clinical diagnosis; duration of the disease; cognition, ambulation, and stair-climbing sub-scores of the modified Barthel index (MBI); manual muscle test (MMT) findings of both lower extremities; functional ambulation category (FAC); and BBS score, were retrospectively analyzed. A multiple linear regression analysis was performed to identify the gait analysis parameters that would significantly correlate with the balance function and other physical performances. In the results, the BBS scores were significantly correlated with the gait speed and step width/height^2^. However, the other gait function measurements, such as the FAC and ambulation and stair-climbing sub-scores of the MBI, were correlated only with the gait speed. Additionally, both the summations of the lower extremity MMT findings and anti-gravity lower extremity MMT findings were correlated with the average swing phase time. Therefore, in the gait analysis, the gait speed may be an important factor in determining the balance and gait functions of the patients with brain lesions. Moreover, the step width/height^2^ may be a significant factor in determining their balance function. However, further studies with larger sample sizes should be performed to confirm this relationship.

## Introduction

Motor deficits are common in patients with brain lesions and often have a great impact on the performance of activities of daily living (ADLs)^1^. Among different motor functions, gait and balance are critical in regaining independent ADL performance^2,3^. Therefore, it is expected that their improvement is the utmost priority in patients with brain lesions. Various measurement tools are used to evaluate the gait and balance functions such as the following: the 6-min walking test^4^, functional ambulation category (FAC)^5^, the Berg balance scale (BBS)^6^, and the modified Rankin scale^7^.

The BBS is a valid and reliable performance-based functional measurement test that evaluates various dynamic and static functional capabilities in the standing and sitting positions^6^. When using the BBS, the participant is asked to perform 14 various functional activities, such as sitting to standing, standing unsupported, reaching forward with an outstretched arm, turning 360°, and alternate stepping in succession onto an 8-inch platform^6^. Using a 5-point ordinal scale, from 0 to 4, the individual is scored based on the ability to maintain and perform the functional task^6^.

However, despite its apparent clinical utility, the BBS has some limitations that may complicate the interpretation of its scores and therefore discourage its use in guiding treatment planning for individuals with brain lesions. First, the BBS has both floor and ceiling effects^8,9^. Second, its inter-rater reliability is lower when assessing post-stroke individuals who score in the mid-range of the scale^8,10^. Third, the BBS score may be a poor predictor of post-stroke falls^8,11–13^. Finally, the score can improve without any actual improvement in the physiological function of standing balance^8,12,13^. Therefore, this score may not reflect the degree of motor function improvement after rehabilitation. Moreover, some individuals can achieve higher BBS scores by utilizing compensatory strategies and without recovering from actual dysfunctions^12^. Hence, individuals who achieve optimal BBS scores may still lack normal balance. Therefore, although it is a well-studied parameter that can evaluate the patients’ balance function, these limitations of the BBS score may limit its use in patients with brain lesions.

Another method in assessing balance impairments is the use of instruments, such as force plates^14^. However, similar to the BBS, certain measurements from force plates may display a combination of impairments and compensatory strategies. One compensatory strategy is to rely on the non-affected limb, while the affected limb is not actively contributing to standing balance control^15^. A measurement of center of pressure displacement, with both feet combined, may not identify such a compensatory strategy. A recent approach to assessing balance impairments in patients with brain lesions is the use of gait analysis^16^. In previous gait analysis studies, patients with brain lesions showed abnormalities in the control of body weight transfer toward the paretic limb side^17,18^. Gait analysis, unlike static foot plate application, is a dynamic evaluation method. The dynamic balance that can be observed may have more influence on the actual ADL performance than static balance^19,20^. Moreover, it is performed during actual gait and can identify compensatory strategies during the gait cycle. Hence, gait speed-related parameters in gait analysis may be correlated with the balance function of patients with brain lesions. However, its validity is not well established. Our hypothesis was that the gait speed- or step width-related parameters may be correlated with the balance function of patients with brain lesions. Therefore, this study aimed to clarify the relationship between the results of gait analysis and balance function, such as the BBS and seek specific measurements that can represent the balance functions of patients with brain lesions. Additionally, we compared other different gait function scale scores, such as the FAC or ambulation sub-score of the modified Barthel index (MBI) with gait analysis measurements.

## Methods

### Participants

We retrospectively reviewed the medical records of patients with brain lesions admitted to our hospital between January 2017 and August 2020.

The inclusion criteria were as follows: (1) age of ≥ 20 years; conditions with brain lesions, such as stroke, traumatic intracranial hemorrhage, or subarachnoid hemorrhage; and ability to walk independently; and (2) gait analysis and clinical assessment of the motor and gait functions, including the MBI, manual muscle test (MMT) of both lower extremities, FAC, and BBS. Conversely, the exclusion criteria were as follows: (1) > 1-week of interval between gait analysis and clinical assessment of the gait and motor functions; (2) patients who cannot walk without the use of walking aids, such as orthoses or a cane; and (3) history of other neurologic or orthopedic conditions that could affect the results of the study. This retrospective study was approved by ethics committee of the Ulsan University Hospital (2020-07-020) and was conducted according to the Declaration of Helsinki for human experiments. Written informed consent was obtained from all participants.

### Clinical assessment

Patient clinical records containing personal data, clinical diagnosis, time of the lesion, cognition (mini mental status examination [MMSE] score)^21^, ambulation and stair-climbing sub-score of the MBI^22^, MMT findings of both lower extremities, FAC, and BBS score were evaluated. All clinical assessments were performed by a physical therapist who was blinded to the gait analysis results.

Using the MMT findings of both lower extremities, we calculated the summation of the findings of both lower extremities (both hip flexor MMT + hip extensor MMT + knee extensor MMT + knee flexor MMT + ankle dorsiflexor MMT + ankle plantar flexor MMT; total, 60) and anti-gravity muscles in the lower extremities (both hip extensor MMT + knee extensor MMT + ankle plantar flexor MMT; total, 30)^23^. All clinical assessments were conducted by the patients’ primary physical therapist within 7 days of the gait analysis. All physical therapists performed the clinical assessments while blinded to the results of the gait analysis.

### Gait analysis

A computer-based instrumented gait analysis system (Walkway MG-1000, Anima, Japan) was used as the gait analysis system herein^24^. This gait analysis system can measure the spatiotemporal parameters of gait from on/off signals between the patients’ foot and the surface of the sensors at a sampling frequency of 100 Hz. The width and length of the walkway system are 0.82 m and 4.8 m, respectively^24^. In the walkway system, data were obtained and processed using the software embedded in the system.

### Experimental procedure

The participants were asked to wear short pants, get on a walking path, and walk barefoot along a 12-m straight line, including 3.5 m in front of the measured walking path and 3.5 m beyond the end of the walking path. No patient used braces or canes. Each participant performed one trial at a comfortable speed in a subjective manner^24^. The patients were asked to walk with their eyes facing forward, and their arms swinging naturally at the sides of the body. A physical therapist with > 10 years of experience performed the gait analysis of all the included patients.

### Gait analysis parameters

As the temporal parameters, the stance phase time (s), swing phase time (s), double stance phase time (s), ratio of the stance phase during the total gait cycle (%), ratio of the swing phase during the total gait cycle (%), ratio of the double stance phase during the total gait cycle (%), and stride time (s) were measured. As the distance parameters, the stride length (m), step length (m), and step width (m) were measured. Additionally, the gait angle (°), toe-out angle (°), gait speed (m/s), and cadence (step/min) were measured.

The gait angle was defined as the angle carried from the connected line of both heels between the right and left feet against the forward direction. The toe-out angle was defined as the open angle of a toe against the forward direction^25^.

Except for the stride length, stride time, gait speed, and cadence, the parameters were measured for each right and left lower extremity. For statistical analysis, we additionally calculated the average value of the parameters of the left and right sides, such as the average stance phase time (s), average swing phase time (s), average double stance phase time (s), average ratio of the stance phase during the total gait cycle (%), average ratio of the swing phase during the total gait cycle (%), average ratio of the double stance phase during the total gait cycle (%), average step length (m), average step width (m), average gait angle (°), and average toe-out angle (°). To compensate for differences in the patients’ height, we also calculated the gait speed/height, gait speed/height^2^, stride length/height, step length/height, step length/height^2^, stride length/height^2^, step width/height, step width/height^2^, stride time/height, and stride time/height^2^.

### Statistical analysis

The Kolmogorov–Smirnov test was used to determine whether the data conformed to a normal distribution. To identify the gait analysis parameters that would significantly correlate with the motor function (FAC, ambulation sub-score of the MBI, and BBS score), we performed a multiple linear regression analysis. To identify which variables would be affected by multicollinearity and the strength of the correlation, we tested for multicollinearity with variance inflation factors (VIFs). Multicollinearity may be present when the VIF is > 5 to 10. Thereafter, multiple linear regression tests were re-performed after removing parameters with multicollinearity. Statistical analyses were performed using SPSS (SPSS Inc., Chicago, IL).

## Results

### Patient characteristics

In this study, 77 patients with brain lesions (54 men and 23 women, 20–72 years of age, 139–185 cm in height, and 43–99 kg in weight) were investigated. Among them, 56 patients had ischemic stroke lesions; 8 patients had hemorrhagic stroke lesions; and 13 patients had traumatic brain injury. Of the patients, 64 had hemiparesis, and 13 had quadriparesis. All patients were admitted in the acute care hospital and underwent physical therapy of 1–2 sessions (30 min per one session) a day when performing gait analysis. The results of the clinical assessments and gait analysis are presented in Tables 1 and 2, respectively. The mean height, body weight, disease duration, total MBI, and MMSE scores were 165.17 ± 8.24 cm, 70.0 ± 12.69 kg, 34.40 ± 116.92 days, 80.67 ± 19.99, and 25.16 ± 6.68, respectively (Table 1). The average values of the summation of the total test scores were 55.72 ± 4.36 for the lower extremity muscle and 27.44 ± 2.76 for the anti-gravity lower extremity muscles (Table 2).

**Table 1:** Clinical and demographic characteristics of included patients with brain lesion.

	Patients
Age (years)	61.92 ± 16.78
Sex (M:F)	54:23
Etiology (ischemic:hemorrhagic:traumatic)	56:8:13
Type (hemiparesis:quadriparesis)	64:13

*M:F* male:female.

**Table 2:** The temporo-spatial parameters of gait in patients with brain lesions.

	Patients
Height (cm)	165.0 (139.0–170.0)
Weight (kg)	65.0 (56.05–75.0)
disease duration (days)	16.0 (12–29.5)
total MBI score	83.50 (68.5–96.0)
MMSE	28.0 (26.0–29.0)
total LE muscles MMT score	54.0 (54.0–60.0)
total antigravity LE muscles MMT score	27.0 (27.0–30.0)
Gait speed (m/s)	0.47 (0.37–0.62)
Total step width (m)	0.52 (0.42–0.57)
Average stance phase time (s)	1.07 (0.88–1.26)
Average swing phase time (s)	0.45 (0.38–0.50)
Average double limb support time (s)	0.29 (0.22–0.36)
Cadence (step/min)	78.17 (70.98–90.60)

Median (interquartile range), *MBI* modified Barthel's index, *MMSE* mini mental status examination, *LE* lower extremity, *MMT* manual muscle test.

### Causal relationship between the balance function and gait analysis parameters

**Figure 1:**
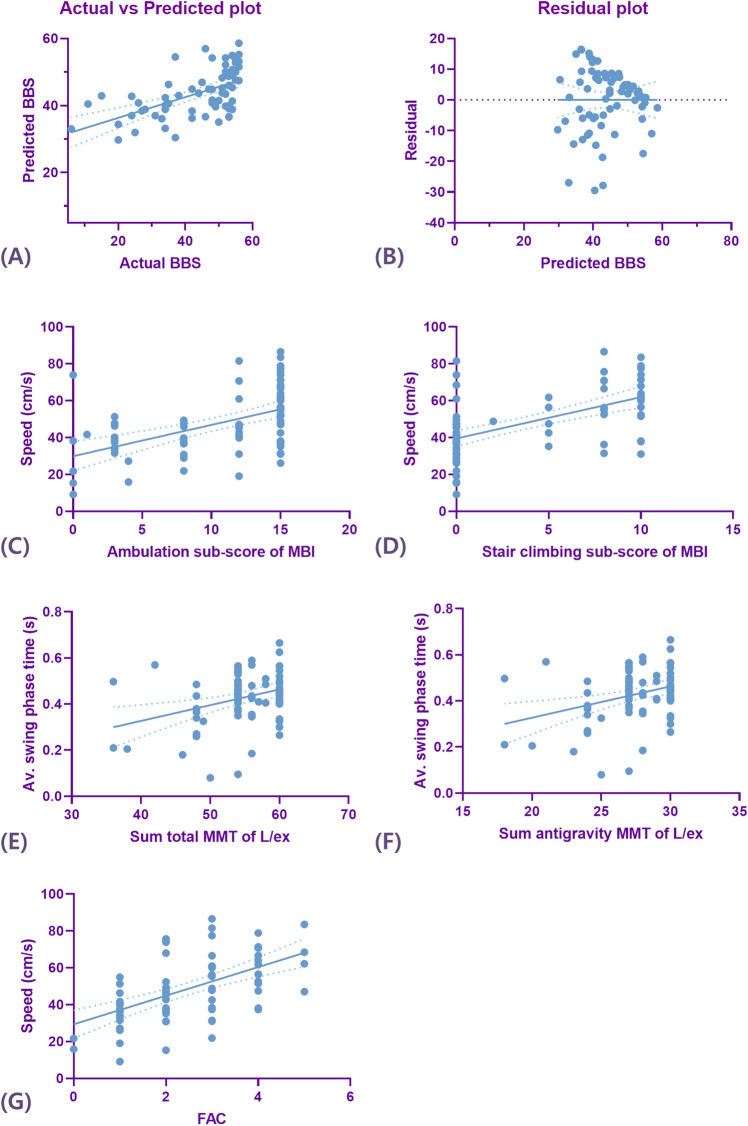
The correlations between the Berg balance scale (BBS) score (**A**) and temporo-spatial parameters of gait (**B**); between the ambulation sub-score of the modified Barthel index (MBI) and temporo-spatial parameters of gait (**C**); between the stair-climbing sub-score of the MBI and temporo-spatial parameters of gait (**D**); between the total summation of the manual muscle test (MMT) score of the lower extremities and temporo-spatial parameters of gait (**E**); (between the summation of the MMT score of the anti-gravity muscles (hip extensor, knee extensor, and ankle plantar flexor muscles) of the lower extremities and temporo-spatial parameters of gait (**F**); and between the functional ambulation category (FAC) and temporo-spatial parameters of gait (**G**).

To evaluate the causal relationship between balance function and gait analysis parameters, the BBS score was used in multiple linear regression analysis. In the multiple linear regression analysis, the gait speed and average step width/height^2^ showed a significant correlation with the BBS score (*p* < 0.05) (Table 3, Fig. 1). However, the speed/height and speed/height^2^ showed multicollinearities; thus, they were removed from the multiple linear regression analysis. The equation for the BBS score and the gait analysis parameters was as follows:

$$ {\text{BBS score}} = 36.63 + \left( {0.55 \times {\text{gait speed }}\left[ {\text{cm/s}} \right]} \right) - \left( {1.05 \times {\text{average step width }}\left[ {{\text{cm}}} \right]/{\text{height}}^{2} \left[ {{\text{cm}}^{2} } \right]} \right) $$

**Table 3:** Multiple linear regression analysis for assessing temporo-spatial parameters of gait in predicting of balance and gait function.

Dependent variable	Independent variables	R^2^	Beta coefficient	Standard error	Odd ratio (95% CI)	*p* value
BBS	Gait speed	0.318	0.756	0.089	0.369–0.726	< 0.001
	Av. step width/height^2^	0.386	− 0.325	0.400	− 1.853–0.252	0.011
FAC	Gait speed	0.305	0.552	0.007	0.026–0.053	< 0.001
Ambulation MBI	Gait speed	0.259	0.509	0.030	0.093–0.213	< 0.001
Stair climbing MBI	Gait speed	0.319	0.565	0.024	0.094–0.190	< 0.001
Total MMT sum	Av. swing phase time	0.112	0.335	5.336	5.817–27.076	0.003
Antigravity MMT sum	Av. swing phase time	0.108	0.328	2.636	2.685–13.187	0.004

*BBS* Berg balance scale, *FAC* functional ambulation category, *MBI* modified Barthel index, *MMT* manual muscle test, *Av* average.

### Causal relationship between the other physical performances and gait analysis parameters

In all the multiple linear regression analyses, the speed/height and speed/height^2^ showed a multicollinearity; thus, they were removed from such analyses.

Between the ambulation sub-score of the MBI and gait analysis parameters, only the gait speed showed a significant correlation with the ambulation sub-score of the MBI (*p* < 0.001) (Table 3). The equation for the ambulation sub-score of the MBI and the gait analysis parameters was as follows:

$$ {\text{Ambulation sub}} - {\text{score of the MBI}} = 3.41 + \left( {0.15 \times {\text{gait speed }}\left[ {\text{cm/s}} \right]} \right) $$

Between the stair-climbing sub-score of the MBI and gait analysis parameters, only the gait speed showed a significant correlation with the stair-climbing sub-score of the MBI (*p* < 0.001) (Table 3). The equation for the stair-climbing sub-score of the MBI and the gait analysis parameters was as follows:

$$ {\text{Stair - climbing sub}} - {\text{score of the MBI}} = - 2.99 + \left( {0.14 \times {\text{gait speed }}\left[ {\text{cm/s}} \right]} \right) $$

Between FAC and gait analysis parameters, only the gait speed showed a significant correlation with the FAC (*p* < 0.001) (Table 3). However, the speed/height and speed/height^2^ showed a multicollinearity; thus, they were removed from the multiple linear regression analysis. The equation for the FAC and the gait analysis parameters was as follows:

$$ {\text{FAC}} = 0.58 + \left( {0.39 \times {\text{gait speed }}\left[ {\text{cm/s}} \right]} \right) $$

Between the MMT findings of the lower extremities and gait analysis parameters, only the average swing phase time showed a significant correlation with the summation of the total MMT scores of the lower extremities (*p* < 0.05) (Table 3). The equation for the summation of the total MMT scores of the lower extremities and the gait analysis parameters was as follows:

$$ {\text{Summation of the total MMT scores of the lower extremities}} = 47.79 + \left( {16.45 \times {\text{average swing phase time }}\left[ {\text{s}} \right]} \right) $$

Moreover, only the average swing phase time showed a significant correlation with the summation of the MMT scores for the anti-gravity muscle of the lower extremities (*p* < 0.05) (Table 3). The equation for the summation of the MMT score for the anti-gravity muscle of the lower extremities and the gait analysis parameters was as follows:

$$ {\text{Summation of the MMT score for the anti}} - {\text{gravity muscle of the lower extremities}} = 24.04 + \left( {7.94 \times {\text{average swing phase time }}\left[ {\text{s}} \right]} \right). $$

## Discussion

The study showed that the BBS scores were significantly correlated with the gait speed and step width/height^2^. However, the other gait function measurements, such as the FAC and ambulation and stair-climbing sub-scores of the MBI, were correlated only with the gait speed. This difference may be because the other assessment tools, such as the MBI or FAC, are categorical. For example, of steps 1–5 for the FAC, only patients in steps 3, 4, and 5 will be able to undergo gait analysis. Additionally, of steps 1–5 for the ambulation or stair-climbing sub-score of the MBI, only patients in steps 4 and 5 will be able to undergo gait analysis. Therefore, the patients who can complete gait analysis would mostly be classified within 2–3 categories. Conversely, the BBS is a point assessment tool. Therefore, owing to its diversification, the step width/height^2^ could additionally be correlated with the BBS score.

Previously, Kobayashi et al.^26^ have reported similar results; the BBS score showed correlations with the gait speed, step length of the affected side, and stride length. However, all these three parameters were related to the gait speed. In contrast, our study showed additional correlations with the step width/height^2^. This difference may be attributed to the difference in the patient group and subject number. In their study, only eight patients with stroke wearing ankle–foot orthoses were enrolled. Moreover, in contrast with our study, several parameters that can be affected by the height or leg length were not corrected by the height or leg length. Therefore, the small number of participants and the non-correction with the height or leg length may have caused the difference with our results.

One interesting finding herein was that both the summations of the lower extremity MMT scores and the anti-gravity muscle MMT score of the lower extremities were correlated with the average swing phase time. We also evaluated the relationship between balance function and the results of gait analysis in hemiplegic patients with brain lesions. In hemiplegic patients, unlike whole patients, both the summation of the hemi-side lower extremity MMT scores and the summation of the antigravity muscle MMT score in hemi-side lower extremity were correlated with the hemi-side step length/height^2^ (Supplementary 1). However, in hemiplegic patients with brain lesions, the BBS score was correlated with gait speed (Supplementary 1). In previous studies, gait speed also has shown significant correlations with functional scale, such as the stroke impact scale (SIS) or physical domain of quality of life in patients with stroke^27,28^. However, the correlation with balance function was never studied. Interestingly, unlike the balance and gait functions, the lower extremity muscle strength did not show a correlation with the gait speed or step width, which may be associated with not only muscle strength but also other factors, such as proprioception of the lower extremities^29,30^.

Our study has several limitations. First, the patient group was heterogeneous. Further, the location of the brain lesions differed among the patients. The term brain lesion itself includes ischemic and hemorrhagic stroke lesions, in addition to traumatic intracranial hemorrhage. Therefore, we also evaluated the relationship between balance function and the results of gait analysis in hemiplegic patients with brain lesions. Interestingly, in both the whole patient group and the hemiplegic patient group, balance function was correlated with gait speed. However, further studies on a single lesion etiology are needed in the future to evaluate the relationship between balance function and gait analysis results. Second, patients with severely compromised gait function were excluded from this study. The participants were mandated to complete gait analysis; therefore, those with severe lower extremity motor dysfunction or balance dysfunction were not able to participate. Thus, the patients who had a balance function sufficient to measure the BBS score but were unable to walk were excluded from this study. As such, the study results cannot be generalized to all patients with brain lesions. Third, the patients underwent the gait analysis without any assistance, such as an ankle–foot orthosis or a cane. Therefore, our research results may not reflect the patients’ balance or gait function well if they improve their gait function with the help of an ankle–foot orthosis or a cane. Lastly, the spasticity was not considered in this study. Owing to the retrospective design of the study, spasticity was not sufficiently evaluated. Therefore, further studies are necessary to evaluate the effect of spasticity on balance function in patients with brain lesions.

## Conclusion

In the gait analysis, the gait speed may be an important factor in determining the balance and gait functions of the patients with brain lesions. Moreover, the step width/height^2^ may be a significant factor in determining their balance function. The balance function in patients with brain lesions plays a critical role in their ADL performance. Therefore, accurate evaluation of an individual’s balance function is important in predicting the prognosis and planning post-discharge treatment. Although the BBS is a well-known, well-proven evaluation tool for the balance function, it has its limitations. In such cases, using gait analysis parameters would be very helpful in assessing the current balance function status and planning further treatment strategies. Further studies with larger sample sizes should be performed to confirm this relationship.

## Supplementary Information

Supplementary Information.
