# Supplementary material for: Evaluation of balance functions using temporo-spatial gait analysis parameters in patients with brain lesions
Source: Sci Rep. 2021 Feb 2;11:2745. doi: 10.1038/s41598-021-82358-2 (PMC7854662; doi:10.1038/s41598-021-82358-2)
Supplement: Supplementary file 1 — Supplementary Information. [file 41598_2021_82358_MOESM1_ESM.docx]

**Evaluation of Balance Functions Using Temporo-Spatial Gait Analysis Parameters in Patients with Brain Lesions**

**Authors**

Byung Joo Lee, M.D^1^, Na-Young Joo, M.S.^2^, Sung Hyun Kim, M.D.^2^, Chung Reen Kim, M.D. ^2^, Dongseok Yang, M.D.^2^, Donghwi Park, M.D.^2*^

**Affiliations**

^1^Department of Rehabilitation medicine, Daegu Fatima Hospital, Daegu, Republic of Korea

^2^Department of Physical Medicine and Rehabilitation, Ulsan University Hospital, University of Ulsan College of Medicine, Ulsan

*Correspondence: Donghwi Park, M.D., Department of Physical Medicine and Rehabilitation, University of Ulsan College of Medicine, Ulsan University Hospital, 877 Bangeojin sunhwando-ro, Dong-gu, Ulsan, 44033, Republic of Korea (e-mail: [bdome@hanmail.net](mailto:bdome@hanmail.net)) TEL : +82-52-250-7222 FAX : +82-52-250-7228

**Supplementary 1.** Multiple linear regression analysis for assessing temporo-spatial parameters of gait in predicting of balance and gait function in hemiplegic patients with brain lesions.

| Dependent variable | Independent variables | R^2^ | Beta coefficient | Standard error | Odd ratio (95% CI) | P value |
| --- | --- | --- | --- | --- | --- | --- |
| BBS | Gait speed | 0.494 | 0.353 | 0.077 | 0.199-0.507 | <0.001 |
| Total MMT sum of the paretic lower extremity | Paretic side Step length/Height^2^ | 0.382 | 0.406 | 0.098 | 0.211-0.600 | <0.001 |
| Antigravity MMT sum of the paretic lower extremity | Paretic side Step length/Height^2^ | 0.384 | 0.201 | 0.049 | 0.105-0.298 | <0.001 |

BBS, Berg Balance Scale; FAC, Functional Ambulation Category; MBI, Modified Barthel Index; MMT, Manual Muscle Test; Av, Average
